# Transmission Dynamics and Effectiveness of Control Measures during COVID-19 Surge, Taiwan, April–August 2021

**DOI:** 10.3201/eid2810.220456

**Published:** 2022-10

**Authors:** Andrei R. Akhmetzhanov, Hao-Yuan Cheng, Natalie M. Linton, Luis Ponce, Shu-Wan Jian, Hsien-Ho Lin

**Affiliations:** National Taiwan University College of Public Health, Taipei, Taiwan (A.R. Akhmetzhanov, L. Ponce, H.-H. Lin);; Taiwan Centers for Disease Control, Epidemic Intelligence Center, Taipei (H.-Y. Cheng, S.-W. Jian);; Hokkaido University Graduate School of Medicine, Sapporo, Japan (N.M. Linton)

**Keywords:** COVID-19, Transmission dynamics, control measures, Taiwan, SARS-CoV-2, coronavirus disease, severe acute respiratory syndrome coronavirus 2, viruses, respiratory infections, zoonoses, vaccine-preventable diseases, mitigation

## Abstract

An unprecedented surge of COVID-19 cases in Taiwan in May 2021 led the government to implement strict nationwide control measures beginning May 15. During the surge, the government was able to bring the epidemic under control without a complete lockdown despite the cumulative case count reaching >14,400 and >780 deaths. We investigated the effectiveness of the public health and social measures instituted by the Taiwan government by quantifying the change in the effective reproduction number, which is a summary measure of the ability of the pathogen to spread through the population. The control measures that were instituted reduced the effective reproduction number from 2.0–3.3 to 0.6–0.7. This decrease was correlated with changes in mobility patterns in Taiwan, demonstrating that public compliance, active case finding, and contact tracing were effective measures in preventing further spread of the disease.

SARS-CoV-2, the pathogen causing COVID-19, began infecting humans in Wuhan, China, in December 2019. Within 1 year, SARS-CoV-2 spread to nearly all countries, and >178 million infections and 3.7 million deaths were reported by April 2021. Taiwan, an island with 23.8 million inhabitants, reported only slightly more than 1,000 cases by April 2021, despite being located close to the original epicenter of the COVID-19 outbreak. At that time, most infections confirmed in Taiwan were acquired abroad, and <10% were acquired locally. 

The subsequent emergence of more transmissible SARS-CoV-2 variants led to multiple introductions from those traveling to and from Taiwan, initiating cryptic transmissions in the capital city of Taipei and its surroundings in April 2021. Newly detected clusters of the virus led to an explosive growth in cases, and daily reported case numbers reached 200 by mid-May. The sudden increase in cases prompted the government to implement stricter control measures to prevent disease spread, and those measures proved effective in bringing the epidemic under control by the end of July. Those preventive measures included restricting public movement, enforcing compulsory shortening of business hours, implementing work-from-home for nonessential businesses, banning in-restaurant dining, and canceling social and religious gatherings. By October 2021, Taiwan was again reporting 0 cases daily.

The initial clusters of infections in 2021 were linked to international pilots and flight crew members, but the major epidemic hotspots were identified as owners and visitors of tea houses, which are landmarks in some districts of Taipei. Although tea houses in Taipei typically offer tea and other refreshments during the day, some also conduct business in the evening, when the potential for activities that increase risk for the transmission of SARS-CoV-2 (e.g., close physical contact) is greater and timely detection of infections can be hindered (*1*–*3*). In nightlife districts across the city, patrons and staff of tea houses and other establishments often are unwilling to share contact and travel histories with public health officials. Outside of Taipei and New Taipei City, clusters of infections were frequently linked to factories or other production sites, affecting vulnerable social groups such as migrant workers. Some initial clusters were linked to local markets and initiated by vendors traveling to the Taipei area for commercial purposes.

The effective reproduction number, R_t_, has played a pivotal role in evaluating the effectiveness of various public health and social measures (PHSMs) during the COVID-19 pandemic (*4*–*6*). R_t_ is defined as the average number of secondary transmissions caused by a primary case at a given time while interventions, existing immunity, or other mediating factors are present. During the pandemic, R_t_ was used frequently as a data point to inform decision- and policy-making processes, because the value of R_t_ relative to the threshold value of 1 can be interpreted as an indicator for when PHSMs should be implemented, strengthened, or relaxed (*7*,*8*). Among the various PHSMs that might be used, stay-at-home orders, cancelling leisure activities, and restaurant-based interventions were found to be largely ineffective in curbing COVID-19 transmission in the United States (*9*). In contrast, strong social distancing, school closures, and widespread mask-wearing were found to be quite effective in mitigating the spread of COVID-19 in both the United States and elsewhere (*10*–*12*). One study found that only strict (complete) lockdowns could curb the spread of infections and reduce R_t_ to <1 (*5*). However, responses to the virus in Taiwan and Japan demonstrate that less extreme measures (i.e., without the implementation of a complete lockdown) were sufficient in preventing a wide, rampant spread of COVID-19 during the epidemic and returning daily counts to an acceptable level (<10 cases). The government’s response to the surge of COVID-19 cases in Taiwan that began in May 2021 presents a striking example of how public compliance with such less extreme preventive measures successfully quelled a burgeoning epidemic wave.

Among various possible ways to estimate R_t_, the instantaneous reproduction number based on the method of Cori et al. (*13*) has often been used during the COVID-19 pandemic to describe current epidemiologic situations (*14*,*15*) or to forecast future incidence (*16*). Predicting the real-time R_t_ value and accounting for covariates has been recognized as an important step toward the future real-time monitoring of disease spread in different countries (*17*–*21*).

Taiwan reported extremely low numbers of confirmed COVID-19 cases in 2020, offering an example of a relatively efficient prevention strategy against the spread of SARS-CoV-2 (*22*). The government instituted a 4-level system to efficiently contain and mitigate COVID-19 epidemics (Appendix Table 1). Before April 2021, the largest cluster of locally acquired infections had only 22 confirmed cases (*23*). Of the various factors contributing to Taiwan’s early pandemic success, the key components were strict border control, public compliance with untargeted PHSMs (e.g., mask-wearing, proactive case finding, and contact tracing), and use of digital technologies, such as QR codes (*24*). However, the increased transmissibility of subsequent SARS-CoV-2 variants and low levels of vaccine coverage posed significant challenges for COVID-19 containment in Taiwan in 2021. We investigated the effectiveness of the public health and social measures instituted by the Taiwan government during the 2021 COVID-19 surge by quantifying the change in R_t_.

## Methods

### Data Collection

We retrieved line list data from publicly available sources and Taiwan Centers for Disease Control reports (*25*). The combined dataset from these sources contained de-identified case records, including information on symptom onset date (when available), case confirmation date, confirmed date of death, level of severity of the infection (asymptomatic/mild, moderate, severe), and information on residency. The 3 categories of disease severity (mild, moderate, severe) were assigned in accordance with the World Health Organization definition (*26*). We extracted mobility metrics from community reports provided by Google (*27*). The 6 metrics used fell into the following categories: “grocery and pharmacy,” “parks,” “residential,” “retail and recreation,” “transit stations,” and “workplaces.” We quantified each metric by a daily change in the median mobility when compared with the baseline median for the 5-week period January 3–February 6, 2020.

### Estimating Epidemiologic Parameters

We fitted time intervals from symptom onset to case confirmation, onset to severe disease, onset to death, and onset to report of death (as well as from death to report of death) to a mixture of 3 distributions (gamma, Weibull, and log-normal) (*23*). We then fitted the serial interval distribution to left-shifted gamma, Weibull, and log-normal distributions (to account for negative values). We estimated all parameters within a Bayesian framework, using a doubly censored likelihood with right truncation and Markov chain Monte Carlo simulations (*28*,*29*). To improve convergence of the mixture model, we set the mean and SDs to be common to the 3 distributions, as has been proposed for Bayesian model averaging (M. Keller et al., unpub. data, https://doi.org/10.48550/arXiv.1711.10016). We estimated the reporting delay, which is the time from symptom onset to case confirmation, under 2 scenarios: when the distribution was unchanged over time, and when the parameters of the distribution were varied in time (*30*).

We estimated R_t_ using date of symptom onset and date of infection (*13*,*31*). When R_t_ was classified by date of symptom onset, the expected case count on day t was proportional to R_t_ and a convolution of case counts on previous days with the serial interval distribution. When R_t_ was classified by date of infection, the formula had a more complicated form and contained a double convolution, involving the incubation period and profile of infectiousness (*31*,*32*) (Appendix). Because some case records did not contain information on symptom onset date, we back-projected those cases from the date the case was confirmed to a presumptive date of symptom onset, using a time-varied distribution of the reporting delay.

## Results

### Epidemiologic Situation

**Figure 1:**
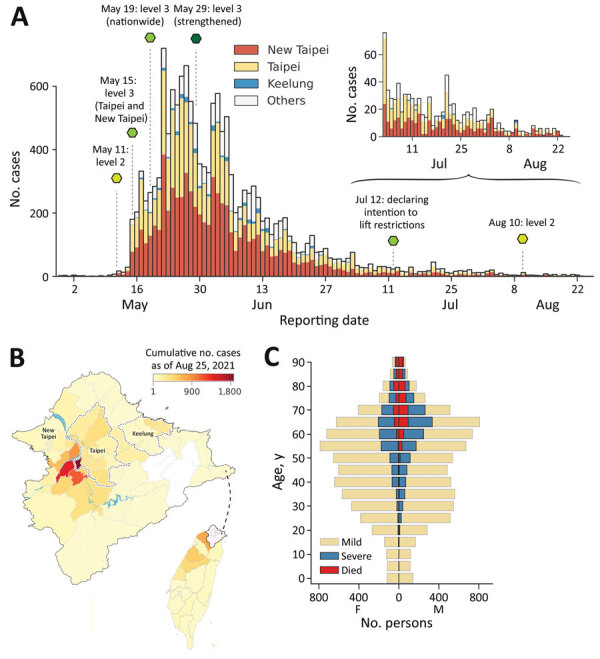
Epidemic wave of COVID-19 in Taiwan, April–August 2021. A) Epidemiologic curve of confirmed COVID-19 cases by reporting date, stratified by geographic area. Dashed lines and hexagons indicate timing and description of major public health and social measures; variation in hexagon colors shows relative strictness of measures, ranging from light to dark green. B) Geographic distribution of cases. The colormap indicates the cumulative number of cases confirmed by August 25, 2021, at district level for Taipei, New Taipei City, and Keelung and at county level for all other areas (indicated in gray in panel A). Inset shows location of enlarged area in Taiwan. C) Age pyramid of confirmed cases specified by known severity status or death. Age and spatial distribution of fatalities is shown in Appendix Figure.

Little to no local transmission of SARS-CoV-2 was reported in Taiwan before April 2021. Vaccine coverage was also arbitrarily low (<1%) at that time. There were, however, multiple clusters of infections during the latter half of April 2021, followed by a wave of COVID-19 cases at the beginning of May 2021 (Figure 1, panel A). A total of 14,442 cases associated with the epidemic wave were confirmed by August 25, 2021, including5,029 (34.8%) persons who were asymptomatic at the time of testing and 3,093 (21.4%) persons recognized as having severe disease. Among patients requiring hospitalization, 238 (1.6%) had nonsevere pneumonia, 2414 (16.7%) had severe pneumonia, and 441 (3.1%) had acute respiratory distress syndrome (Table). A total of 779 persons (5.4%) died during the epidemic wave. Most (701, 90%) of the deceased patients had known underlying chronic conditions. Eight additional deaths among patients in the study population were unrelated to SARS-CoV-2 infection.

**Table:** Demographic and clinical characteristics of persons with confirmed COVID-19 cases, by geographic region, Taiwan, April 23, 2021–August 25, 2021*

	No. (%)			
Characteristic	Taiwan	Taipei	New Taipei City	Other counties
Age group				
<17	845 (5.9)	225 (4.6)	410 (6.0)	210 (7.7)
17–34	2,660 (18.4)	642 (13.2)	1,168 (17.0)	850 (31.0)
15–64	7,489 (51.9)	2,629 (54.2)	3,656 (53.3)	1,204 (44.0)
>64	3,448 (23.9)	1,354 (27.9)	1,620 (23.6)	474 (17.3)
Sex				
F	7,149 (49.5)	2,502 (51.6)	3,387 (49.4)	1,260 (46.0)
M	7,293 (50.5)	2,348 (51.6)	3,467 (49.4)	1,478 (54.0)
Severity				
Mild/asymptomatic	11,349 (78.6)	3,807 (78.5)	5,309 (77.5)	2,233 (81.6)
Severe	3,093 (21.4)	1,043 (21.5)	1,545 (22.5)	505 (18.4)
Known to be symptomatic				
No	5,037 (34.9)	1,710 (35.3)	2,193 (32.0)	1,134 (41.4)
Yes	9,405 (65.1)	3,140 (64.7)	4,661 (68.0)	1,604 (58.6)
Total	14,442	4,850 [33.6]	6,854 [47.5]	2,738 [19.0]

*Parentheses indicate a columnwise fraction of cases within each group. Brackets indicate a rowwise proportion of cases.

The median age of persons with confirmed cases was 51 years; 23.9% were >65 years of age, 51.9% 45–64 years of age, 18.3% 18–44 years of age, and 5.9% <18 years of age. Only 0.4% of patients <18 years were categorized as having moderate disease, and half (50.3%) of these younger patients were reported as asymptomatic at the time of testing. In contrast, 46.6% of those >65 years of age experienced moderate symptoms, and 22.3% were asymptomatic at the time of testing. The median age of patients who died was 72 years, and 79.8% of deaths were reported among those >65 years of age. Men accounted for most deaths (63.5%). Geographically, a substantial portion of the infections (1,874 cases, 13.0% of the total) were confirmed among residents of Wanhua District in Taipei (Figure 1, panel B).

The median time from date of symptom onset to date of case confirmation was estimated at 3.0 days (95% CI 0.7–11.9 days). The time required for disease progression from symptom onset to severe disease was an average of 7.7 days (95% CI 2.1–28.5 days). Death was observed, on average, 13.3 days after symptom onset (95% CI 1.1–92.4 days). Deaths were reported an average of 3.5 days thereafter (95% CI 1.0–12.3 days). 

### R_t_ and Efficiency of PHSMs

**Figure 2:**
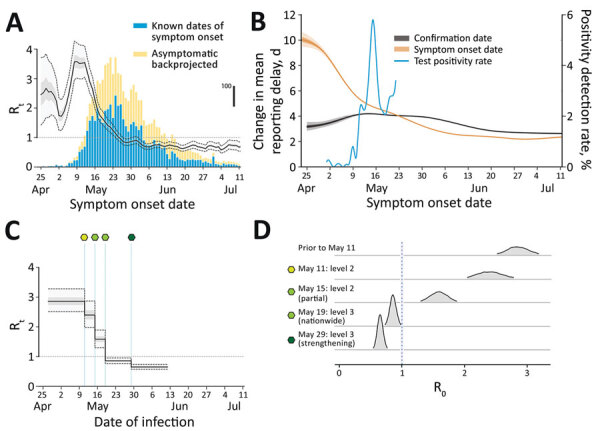
Comparison of R_t_ inferred by infection date with R_t_ by symptom onset date during epidemic wave of COVID-19 in Taiwan, April–August 2021. A) R_t_ by infection date (overlay) is notably shifted to the left compared with symptom onset date. Black line indicates mean; light gray shading indicates interquartile range; dotted lines indicate 95% CI. Bars indicate the nowcasted daily incidence of COVID-19 cases; vertical scale is indicated by thick black line on the right. B) Change in the mean reporting delay, which is the time between symptom onset date and confirmation date, over time, characterized by either the date of symptom onset (orange) or by confirmation date (black). Dark gray shading indicates IQR; light gray shading indicates 95% CI. The blue line indicates the test positivity rate that peaked around May 16 (axis on the right). C, D) The estimated R_t_ by date of infection, linked to public health and social measures (green-shaded hexagons, as defined in panel D). R_t_, effective reproduction number.

When quantifying R_t_ by date of symptom onset, we noted that the value remained relatively stable, with values of ≈2–3 before the surge of COVID-19 cases reported around May 10, 2021 (Figure 2, panel A). We estimated the median posterior value of R_t_ to exceed 3 during the first week of May, likely because of cryptic community transmission; confirmed cases with symptom onset in the first week of May had prolonged reporting delays of nearly 10 days (Figure 2, panel B, orange line), and later cases generally had shorter reporting delays of ≈3–4 days. The reporting delay quantified by date of case confirmation peaked around May 16 (Figure 2, panel B, gray line). The test-positivity rate for SARS-CoV-2 also reached its highest around the same dates (Figure 2, panel B, blue line). These results indicate that cases with earlier symptom onset dates had longer reporting delays compared with subsequent cases and serve as an indicator of persistent cryptic transmission of SARS-CoV-2 in the community between the end of April and the beginning of May 2021.

Next, we quantified the effective reproduction number by infection date and tied it to PHSMs (Figure 2, panels C, D). Taiwan adopted a 4-tier system of restrictions ranging from level 1 at the lowest to level 4 at the highest (Appendix Table 1). Level 2 restrictions began on May 11, 2021; level 3 restrictions began in Taipei and New Taipei City on May 15 and then expanded to the rest of Taiwan on May 19. Level 3 measures were further strengthened on May 29. We estimated the posterior mean R_t_ in the early stage of the epidemic—before level 2 restrictions began—at 2.85 (95% CI 2.51–3.26). Implementation of level 2 measures on May 11 was followed by a slightly decreased mean of 2.40 (95% CI 1.99–2.86), and level 3 measures in Taipei City and New Taipei City on May 15 further decreased the mean value to 1.59 (95% CI 1.30–1.90). Nonetheless, these measures were insufficient to bring the R_t_ consistently below 1. Only after level 3 measures were expanded to all of Taiwan on May 19 did the mean R_t_ decrease to below 1 (0.86 [95% CI 0.76–0.95]). R_t_ then dropped even further when those measures were strengthened on May 29 by prohibiting dine-in services and setting up a work-from-home order (0.65 [95% CI 0.57–0.74]).

These estimates prompted our further investigation into why the initial set of level 3 measures implemented on May 15 for Taipei and New Taipei City and on May 19 nationwide were insufficient to bring R_t_ substantially below 1. We estimated R_t_ by infection date using 2 different functions of time. First, R_t_ was modeled by a piecewise constant function of time with equidistant time windows (e.g., 5 or 7 days). Second, the change in R_t_ was correlated with the observed change in community mobility across 6 different community metrics (see Methods).

**Figure 3:**
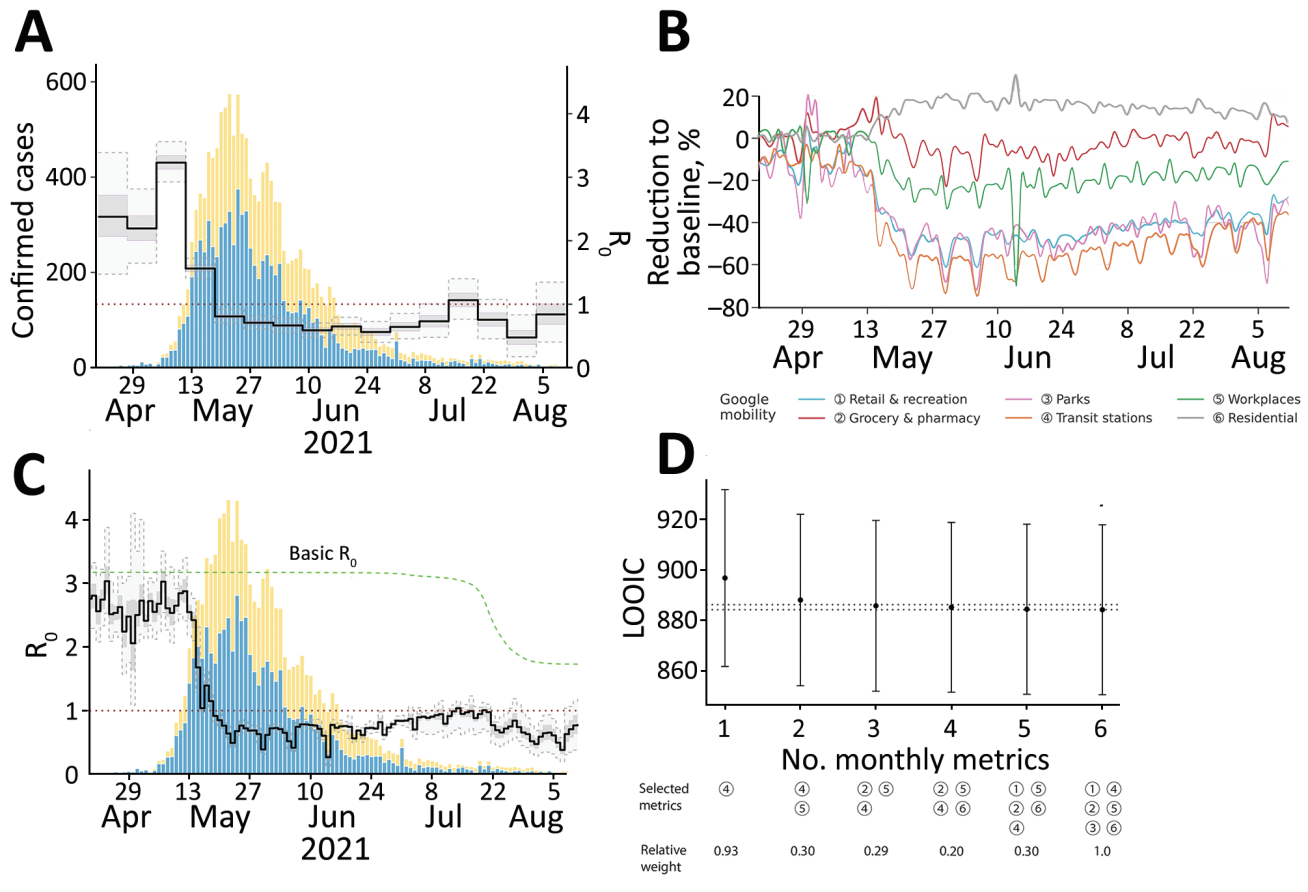
R_t_ by infection date and its link to mobility patterns for epidemic wave of COVID-19 in Taiwan, April–August 2021. A) The change in R_t_ modeled by a piecewise constant function with a 7-day time window. B) The R_t_ inferred based on monotonically decreasing basic reproduction number (green) and 6 mobility metrics. C) The temporal dynamics of mobility metrics. D) Comparison of different models based on LOOIC values under a restricted number of mobility metrics (numbers defined in panel B). The legend indicates the set of metrics with highest probability of selection shown by relative weight. Dashed lines contain the region where the change in LOOIC values does not exceed 2 from the minimum, implying a relatively equivalent fit to the data; error bars indicate SD. The blue and yellow bars in A and C are the same as in Figure 2, panel A. LOOIC, leave-one-out information criteria; R_t_, effective reproduction number.

When we modeled R_t_ using a piecewise constant function of time, we observed a pattern similar to that of R_t_ by date of symptom onset, except that the pattern was time-lagged (compare Figure 3, panel A, and Figure 2, panel A). The temporal pattern also resembled the change in various mobility metrics over time (compare Figure 3, panel A, and Figure 3, panel B). However, the posterior mean of R_t_ did not increase after July 12, even though some mobility metrics previously recognized as important for explaining the transmission potential of COVID-19 (*17*) (e.g., retail and recreation, transit stations, and workplaces) continued to increase over time. To address this contradiction, we theorized that the basic reproduction number (R_0_) changed over time. The time-variability of R_0_ represented the proxy measure of changing contact rate of infected and susceptible individuals over time and served as an indicator of PHSMs, including the voluntary changes in public behavior (*33*). When we defined it by a monotonically decreasing sigmoidal function over time, the corresponding model fit the data better. We compared a model with a time-varied R_0_ with a model with a constant R_0_ using a “leave-one-out” information criteria (LOOIC), which is used in Bayesian frameworks for model selection (*34*). The model with a time-varied R_0_ had a lower median LOOIC value (884.2) compared with that of the model that used a constant R_0_ (899.6) (Appendix Figure 5). The fit resulted in the change point of R_0_ on approximately July 19, and R_0_ decreased from a median of 3.17 at the beginning of the epidemic to 1.72 at the end of the epidemic (defined as August 14), a 46% reduction.

We additionally investigated the association of different mobility metrics with R_t_. The model with only 3 mobility metrics showed a fairly indistinguishable data fit compared to models with 4 to 6 mobility metrics, and the difference in LOOIC values was <2 (ΔLOOIC ≤1.56). By sequentially fitting the models with 1, 2, and 3 metrics, we identified that the most significant metrics describing the individual mobility were transit stations, workplaces, and grocery stores and pharmacies (Figure 3, panel D).

**Figure 4:**
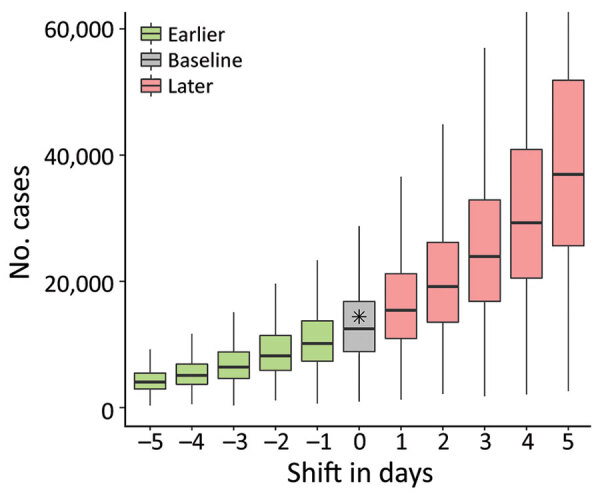
Epidemic wave of COVID-19 in Taiwan, April–August 2021. Model shows impact on epidemic size (by August 14, 2021) of a delay in implementing level 3 prevention measures (Appendix Figure 2) or of implementing them earlier. Horizontal line within boxes indicate medians; box tops and bottoms indicate interquartile ranges; whiskers indicate 95% CIs. Gray box indicates baseline scenario; asterisk indicates observed data.

We investigated counterfactual scenarios wherein level 3 measures had been implemented either earlier or later than the actual May 15 date (Figure 4). If the level 3 measures had been delayed by just 3 days, the size of the epidemic on August 14 likely would have been double that of the baseline scenario (23,900 cases [95% CI 7,900–61,500)] vs. 12,500 cases [95% CI 4,000–29,800]) or the actual case count (14,400). Beginning level 3 measures 3 days earlier likely would have resulted in only 6,400 cases (95% CI 2,200–15,600) (Appendix Figure 6). Varying the date of level 3 implementation revealed a nonlinear, exponential-like relationship whereby a longer delay would accelerate the increase in the final epidemic size.

## Discussion

In this study, we analyzed the spread of SARS-CoV-2 in Taiwan during April–August 2021 and quantified the effectiveness of PHSMs implemented by the government. Initial COVID-19 cases had longer reporting delays, and there was a higher test-positivity rate at the beginning of the outbreak (Figure 1). Shortening of the reporting delay over time (Figure 2, panel B) indicated better management of the outbreak in later periods. Our results also showed that implementing stricter PHSMs on May 29, 2021 (Appendix Table 2), was followed by R_t_ falling below 1. We conclude that the timing of introduction of PHSMs by the government was judicious, and postponement by >3 days would have likely more than doubled the final size of the outbreak.

Because the number of cases grows exponentially at the beginning of an outbreak, delaying PHSMs by just 3 days can lead to a significant increase in the disease burden and can double the final epidemic size. Given the indications that the healthcare system of Taiwan was close to being overwhelmed with COVID-19 patients in mid-May, the actual timing of level 3 measures on May 15 likely prevented an even larger healthcare crisis. Although an earlier introduction of PHSMs could have substantially improved the situation, the low case numbers might have caused some public misunderstanding regarding the necessity of strict prevention measures when there was no evidence of escalating case counts. It is fortunate that the government of Taiwan acted in accordance with the country’s 4-level COVID-19 alert system criteria by implementing stricter PHSMs as soon as possible (Appendix Table 2).

The April–August 2021 epidemic wave was the first such large-scale wave seen in Taiwan. Using Bayesian statistical inference of the effective reproduction number by date of infection, we were able to attribute reductions in R_t_ to the implementation of PHSMs and estimate their effectiveness. The value of R_t_ only fell below 1 (95% CI 0.57–0.74) consistently after the PHSMs were further strengthened. We base this result, however, largely on model assumptions, so the association might be confounded by behaviors not accounted for in the models.

Even assuming only 1 in 5 COVID-19 cases was confirmed, the cumulative number of cases would have reached fewer than 100,000 cases, according to our models. In 2022, however, Taiwan experienced a much larger outbreak associated with the Omicron variant, during which the total number of confirmed cases exceeded 4 million. Given Omicron’s higher transmissibility and greater capacity for evading immunity, coupled with pandemic fatigue and high vaccination coverage of the Taiwanese population (80.2% for the second dose and 60.1% for the booster dose as of May 2, 2022), the government chose to relax PHSMs such as proactive case finding and contact tracing in mid-May 2022. As a result, a direct comparison between the pandemic situation in 2021 we have described and the 2022 Omicron wave is not possible.

Using mobility metrics, which are the proxies of contact rates in different settings, was unable to completely capture the temporal change in R_t_. However, the additional assumption of a simultaneous decrease in R_0_ at later stages of the epidemic adequately explained the observed dynamics. This decrease could likely be a result of higher efficiency in terms of case finding and contact tracing when the number of cases was significantly lower compared with the efficiency in gathering that information at the peak of the epidemic wave.

In regard to study limitations, we did not distinguish fully asymptomatic infections from those that were asymptomatic at the time of testing but became symptomatic later. We also did not account for age, sex, and spatial structures in our framework for estimating R_t_; including those factors could have provided more insight into the transmission dynamics. We also did not distinguish high-risk and low-risk transmission venues in our statistical model, nor did we account for the contribution of superspreading events. We noted, with interest, that the Alpha variant was not the only variant detected among the locally acquired infections during the investigation period. An outbreak associated with the Delta variant also was reported in June 2021, which surfaced in Pingtung County in the south of Taiwan and was contained within 2 weeks. The cluster originated from 2 travelers who returned to Taiwan from Peru and involved a total of 17 cases.

In 2021, Taiwan’s pandemic response demonstrated that, despite low levels of vaccine coverage, containment and elimination of COVID-19 remained feasible. The timely introduction of PHSMs helped Taiwan to avoid healthcare system collapse, and the PHSM strategies employed serve as an example for future outbreaks of emerging and re-emerging infectious diseases. In the case of SARS-CoV-2, the continued evolution of the virus toward higher transmissibility and immune evasion poses a continued threat. It is clear from the 2022 Omicron waves in Taiwan and elsewhere that high levels of vaccine coverage, although offering protection against severe disease, are insufficient in preventing transmission. PHSMs beyond vaccination might become necessary again for future SARS-CoV-2 epidemic waves.

Appendix Additional information about transmission dynamics and effectiveness of control measures during a COVID-19 surge, Taiwan, April–August 2021.
