# Supplementary material for: Transmission Dynamics and Effectiveness of Control Measures during COVID-19 Surge, Taiwan, April–August 2021
Source: Emerg Infect Dis. 2022 Oct;28(10):2051–9. doi: 10.3201/eid2810.220456 (PMC9514361; doi:10.3201/eid2810.220456)
Supplement: Appendix — Additional information about transmission dynamics and effectiveness of control measures during a COVID-19 surge, Taiwan, April–August 2021. [file 22-0456-Techapp-s1.pdf]

Article DOI: https://doi.org/10.3201/eid2810.220456

Transmission Dynamics and Effectiveness of Control Measures during a COVID-19 Surge, Taiwan, April–August 2021

Appendix

Estimation of the serial interval

Estimation of the serial interval was performed by selecting symptomatic infector-infectee pairs from a series of epidemiological investigations conducted by Taiwan Centers for Disease Control (CDC) from the beginning of 2020 through March 2021 (i.e. preceding the epidemic wave under analysis). They were assigned as “certain” or “probable” pairs depending on the strength of the available evidence supporting the epidemiological linkage and directionality of transmission (accurately identifying who was the infector and who was the infectee). Three shifted distributions (gamma, Weibull, and lognormal) were fitted to data on the time between onset of symptoms of infectors and onset of symptoms of infectees for 33 certain infector-infectee pairs and the best-fit distribution was identified using the Bayesian mixture model. In that setting the best-fit distribution was the one with the maximum posterior probability. Inclusion of 25 probable infector-infectee pairs did not significantly alter the inferred estimates. The complete list of infector-infectee pairs used for estimation of the serial interval is provided (Appendix Table 1).

Estimation of within-individual time distributions

The distribution of time intervals from one event of interest to another, including:

- symptom onset to case confirmation,
- symptom onset to severe disease,
- symptom onset to death,
- symptom onset to report of death, and
- death to report of death

were fitted to a mixture of three unimodal (gamma, Weibull, and lognormal) distributions within the framework of a Bayesian mixture model. Each of the three component distributions was defined by the probability density function $f_l(\circ; \theta)$ $(l = 1,2,3)$ that was given a two-dimensional parameter vector $\theta = (\mu, \nu)$, where μ was the mean and ν was the coefficient of variation (CV). Both parameters were common to all three component distributions.

Suppose that the time of the first event for a case k $(k \in K)$ was denoted by o_k and the time of the second event was denoted by c_k. When both times were recorded in the dataset, they were given by their calendar dates, $\mathcal{O}_k$ and $\mathcal{C}_k$, respectively. Both times, o_k and c_k, were assigned to uniform priors of 1-day:

$$o_k \sim \text{Uniform}(\mathcal{O}_k, \mathcal{O}_k + 1 \text{ day}) \tag{1}$$

$$c_k \sim \text{Uniform}(\max(\{o_k, \mathcal{C}_k\}), \mathcal{C}_k + 1 \text{ day}) \tag{2}$$

where $\mathcal{O}_k \le \mathcal{C}_k$ was held for all aggregated records, and $o_k < c_k$ was imposed for simplicity for $\mathcal{O}_k = \mathcal{C}_k$. Because of a non-zero probability of viral RNA detection in a sample from a presymptomatic individual, the condition $o_k < c_k$ can be invalid in general. However, when the alternative model with shifted distributions was verified, we found that it did not improve the data fit, and therefore chose to use the simpler model version.

When the date of the first event, $\mathcal{O}_k$, was not recorded, the case was asymptomatic at the time of testing, $\mathcal{T}_k$. According to the data collection procedures of the Taiwan CDC, the date of testing $\mathcal{T}_k$ is backlogged by 1 day or occurs on the same day as the reporting day $\mathcal{R}_k$ if confirmation of the case represents social significance defined based on expert opinion within the agency. Because the severity status of all confirmed cases was regularly updated by the Taiwan CDC, we noticed that some cases with an initially unknown symptom onset date $\mathcal{O}_k$ became severe at later dates, denoted as $\mathcal{S}_k$. Suppose that two conditions are additionally met: $\mathcal{T}_k \le \mathcal{C}_k$ and $\mathcal{R}_k < \mathcal{S}_\mathrm{k} < \mathcal{C}_k$. The prior (1) can then be replaced with the following prior valid for that subset of cases:

$$o_k \sim \text{Uniform}(\mathcal{T}_k = \mathcal{R}_k - 1 \text{ day}, \mathcal{S}_k + 1 \text{ day}) \tag{3}$$

Otherwise, the cases were omitted from the analysis.

The likelihood was defined by the weighted sum of three components:

$$L_{\Sigma}(\theta; D = \{o_k, c_k\}) = \sum_{l=1}^{3} w_l L_l(\theta; D) \tag{4}$$

$$L_l(\theta; D) = \prod_{k \in K} \frac{f_l(c_k - o_k; \theta)}{F_l(T - o_k; \theta)} \tag{5}$$

where $F_l(\circ; \theta)$ is the cumulative distribution function of $f_l(\circ; \theta)$, and T is the date of the latest release of data by Taiwan CDC, set as 2 p.m. on of August 25, 2021. The probability of selecting the best-fit distribution could be then given by categorical sampling from the three components with relative probabilities $w_l L_l / L_{\Sigma}$ ($l = 1,2,3$).

Time-varied reporting delay

The effective reproduction number, R_t, and case-fatality ratio are often estimated retrospectively when an epidemic has already been declared over, but they can also be estimated in real-time. However, real-time estimation presents unavoidable challenges to researchers (*1,2*). First, snapshots of count data are right-truncated respective to the time of the latest update. In other words, cases that have already been infected but not yet confirmed are not recorded in the data. Unless these truncated cases are accounted for—e.g., by nowcasting—R_t will always show a decreasing trend toward zero just before the latest data cutoff date. Second, a substantial fraction of case records may not contain the necessary information, such as date of symptom onset, as that information might not be required for initial reporting of a case (particularly if the confirmed case definition is based primarily on a positive laboratory test) and thus might only be collected retrospectively. COVID-19 cases can be completely asymptomatic, pre-symptomatic at the time of testing and not followed up later, or have unknown symptom status (e.g., owing to patient noncooperation or a deteriorating health condition). Because missing symptom onset dates can be backprojected from reporting dates, identification of the reporting delay distribution becomes an important quantity to estimate. Hence, we estimated a time-varied reporting delay distribution that allowed us to backproject the symptom onset date for cases with unknown presence of symptoms and to nowcast cases that were not yet reported in Taiwan's COVID-19 epidemic wave in mid-2021.

In our framework, the coefficient of variation (CV) of the reporting delay ν was set constant, but the mean reporting delay μ varied over multiple lengthscales similarly to previous work (*3*). Two lengthscales were used, each characterized by two parameters: W, which defined a long-range lengthscale and ω, which defined a short-range lengthscale. Their implementation

in our statistical framework is described in more detail below. The baseline values for W and ω were set to 10 days and 7 days, respectively (Appendix Figure 1). The sensitivity analysis included other values, $W = \{5 \text{ days}, 15 \text{ days}\}$ and $\omega = \{3 \text{ days}, 11 \text{ days}\}$, demonstrating relative robustness (Appendix Figure 2). A comparison with a piecewise constant reporting delay was also performed (Appendix Figure 3).

For the long-range variation in the reporting delay controlled by the parameter W, the temporal dynamics of its mean were modeled using a cubic B-spline, $\mu(t) = \mu_{\kappa,\Theta}(t)$, such that:

$$\mu_{\kappa,\Theta}(t) = \sum_{n=1}^{N} \vartheta_n B_{n,\kappa}(t, \Theta) \quad (6)$$

where $\kappa = 3$ is the degree of the B-spline, ϑ_n $(n = 1, \dots, N)$ are B-spline coefficients, $\Theta = \{\Theta_\mathrm{m}, m = 0, \dots, M\}$ is a knot sequence composed of equidistant time points $\Theta_\mathrm{m} = Tm/M$, and $N = M + \kappa - 1$ is the total number of the basis spline functions (*4*). The sensitivity of the B-spline function was controlled by the parameter W. The number of splits M was pre-defined as $M = \lfloor T/W \rfloor + 1$, where the operator $\lfloor T/W \rfloor$ was the floor division of T on W. The choice was made to ensure that the distance between the nearest knots, $\delta = T/M$, was not larger than W and had a finite lower bound proportional to W, $W(1 - 1/M) \leq \delta \leq W$.

The likelihood for a short-range variation in the reporting delay controlled by the parameter ω was defined as follows:

$$L_l(\theta_t = \{\mu_{\kappa,\Theta}(t), \nu\}, \{o_k, c_k \mid k \in K(t)\}) = \prod_{k \in K(t)} \frac{f_l(c_k - o_k, \theta_t)}{F_l(T - o_k, \theta_t)} \quad (7)$$

where the set $K(t)$ consisted of all cases whose symptom onset date $\mathcal{O}_k$ (or confirmation date $\mathcal{C}_k$) was within the time window ω: $t - \omega \leq \mathcal{O}_k \leq t + \omega$ (or $t - \omega \leq \mathcal{C}_k \leq t + \omega$), o_k and c_k are the inferred symptom onset and confirmation time:

$$o_k \sim \mathrm{Uniform}(\mathcal{O}_k, \mathcal{O}_k + 1 \text{ day}),$$

$$c_k \sim \mathrm{Uniform}(\max(\{o_k, \mathcal{C}_k\}), \mathcal{C}_k + 1 \text{ day}),$$

as for all records $\mathcal{O}_k \leq \mathcal{C}_k$. The distribution l was chosen among the gamma, Weibull, and lognormal distributions ($l = 1,2,3$). The overall likelihood was present by a sum of three components:

$$L_\Sigma(\theta, D = \{\mathcal{O}_k, \mathcal{C}_k\}) = \sum_{l=1}^{3} w_l \prod_t L_l(\theta_t, \{o_k, c_k \mid k \in K(t)\}) \quad (8)$$

Because the reporting delay could be specified either by the date of symptom onset O_k (a "forward-looking" reporting delay) or by the date of confirmation C_k (a "backward-looking" reporting delay according to notations (*5*)), the two possible reporting delay distributions could lead to different results. The forward reporting delay, indicated by "(o)" in our work, e.g., $f^{(o)}$, describes the situation in which the mean reporting delay was estimated using the left-end symptom onset date. By comparison, the backward reporting delay, indicated by "(c)", e.g., $f^{(c)}$, describes the situation in which the mean reported delay was estimated using the right-end confirmation date. Depending on the concrete application, one form can be preferred over another and would allow different interpretations. For instance, when cases with unknown symptom onset dates were backprojected to their symptom onset dates, the form $f^{(c)}$ could be appropriately used. When the cases were nowcasted to a given confirmation date to predict the number of cases that had not yet been reported, the form $f^{(o)}$ was appropriate. Park and colleagues (*5*) argued that the backward-looking delay distribution is prone to a bias toward lower values when an exponential growth in cases is observed during an epidemic, whereas the forward-looking delay distribution is likely to be representative of the unbiased (intrinsic) delay distribution.

Thus, the reporting delay distribution was modeled based on a mixture of three unimodal distributions (gamma, Weibull, and lognormal). The plausibility of each distribution was determined by its relative weight w_l in the likelihood L_Σ, when the probability of selecting the distribution l was equal to $w_l L_l / L_\Sigma$ (see equations (7) and (8)). The temporary change in the reporting delay was modeled by a time-varied mean reporting delay, whereas the CV of the reporting delay was kept unchanged. The alternative formulation, where the standard deviation (SD) was used instead of the CV and kept constant, did not fit the data, so it was omitted from our analysis. The time variation of the mean reporting delay μ was modeled at two timescales: for the long timescale, the change in $\mu(t)$ was defined by a cubic B-spline function, while for the short timescale the reporting delay distribution was adjusted locally using the likelihood (7). The resulting fit was obtained by combining the two timescales and quantified either by the symptom onset date, denoted by the upper index (o), or by the confirmation date, denoted by the upper index (c).

Effective reproduction number by date of symptom onset

To estimate the effective reproduction number by the date of symptom onset, one needs to know whether the case is asymptomatic or symptomatic. If the case is symptomatic, the symptom onset date should be recorded. However, the extracted dataset was composed of two types of case records—some records contained the symptom onset date and the confirmation date, while others had a blank symptom onset date. For the latter, the asymptomatic status was not definitive, and the record could not differentiate truly asymptomatic and pre-symptomatic cases. The unknown symptom onset dates were backprojected from the case confirmation dates using the reporting delay distribution $f^{(c)}$.

Let i_t be the daily counts of cases with known symptom onset dates at day of symptom onset t, and y_t be the daily counts of cases with unknown symptom onset dates at day of confirmation t. The expected daily count i_t^* that combines both was defined as follows:

$$i_t^* = i_t + \sum_{\tau>0} y_{t+\tau} f_{l,\tau}^{(c)}(\theta_{t+\tau}) \quad (9)$$

where $f_{l,\tau}^{(c)}(\theta_{t+\tau})$ is a discretized reporting delay distribution, $f_{l,\tau}^{(c)}(\theta_t) = F_l^{(c)}(\tau, \theta_t) - F_l^{(c)}(\tau - 1, \theta_t)$ for $\tau = 1,2, \ldots$, and the function $F_l^{(c)}(\circ, \theta_t)$ is the cumulative distribution function of the reporting delay $f_l^{(c)}(\circ, \theta_t)$.

To account for right truncation, the expected counts i_t^* were nowcasted according to (*6*):

$$i_t^{nc} = \frac{i_t^*}{F_l^{(o)}(T-t, \theta_t)} \quad (10)$$

where the function $F_l^{(o)}(\circ, \theta_t)$ is a cumulative distribution function of the forward reporting delay distribution $f_l^{(o)}(\circ, \theta_t)$.

The effective reproduction number by date of symptom onset, $R_t^{(o)}$, was estimated using the renewal process written within the negative binomial likelihood:

$$i_t^{nc} \sim \text{NegBinom}\big(\text{mean} = E(i_t^{nc}), \text{overdisp.} = \phi_{\{1\ldots4\}}\big)$$

$$E(i_t^{nc}) = R_t^{(o)} \sum_{\tau=1}^{t-1} i_{t-\tau}^{nc} \cdot g_\tau \quad (11)$$

where g_τ is the serial interval distribution estimated for Taiwan consisting of the best-fit lognormal distribution with a mean (±SD) of 4.6 ± 3.4 days, which is in line with previous

reports (*7*,*8*). The overdispersion parameter ϕ followed one of the four functional forms: (*i*) $\phi_1 \equiv \text{const}$, which is a commonly used form of the negative binomial distribution, also known as NB2, (*ii*) $\phi_2 \equiv \text{const} \cdot E(i_t^{nc})$, which resembles a quasi-Poisson distribution, also known as NB1, (*iii*) $\phi_3 \equiv \text{const} \cdot (E(i_t^{nc}))^{1/2}$, which presents an intermediate situation between (*i*) and (*ii*), and (*iv*) $\phi_4 \gg 1$, which is a limiting case leading to the Poisson likelihood. Green and colleagues (*9*) previously indicated a better fit of the data when negative binomial likelihood (*i*) is used instead of Poisson likelihood (*iv*). However, other studies (*10*,*11*) have shown that the overdispersion parameter ϕ given by (*iii*) is a better choice than (*i*) or (*ii*). In our framework, all four likelihoods (*i*)–(*iv*) were implemented within the Bayesian mixture model with a similar likelihood as in (8). The best-fit configuration was directly selected from the results of statistical inference.

Because the nowcasted counts i_t^{nc} (10) are expected values and are not integer counts of incidence, the negative binomial likelihood (11) was replaced with the gamma likelihood that matched the first and the second moments (*12*):

$$i_t^{nc} \sim \text{Gamma}(\text{shape} = \alpha_t, \text{rate} = r_t) \qquad (12)$$

where $r_t = \phi_{\{1\ldots4\}}/(\phi_{\{1\ldots4\}} + E(i_t^{nc}))$ and $\alpha_t = E(i_t^{nc}) \cdot r_t$.

Finally, the effective reproduction number was smoothed by calculating the centered rolling average over a 5-day time window (*13*), which implied that the value $R_t^{(o)}$ on day *t* was estimated using five sequential values of the nowcasted counts on days $\{t-2, t-1, t, t+1, t+2\}$.

Effective reproduction number by date of infection

The number of infections j_t on day t was generated by nowcasted cases with symptom onset dates on either side of that day and was proportional to the effective reproduction number by the date of infection $R_t^{(i)}$. Framed within the renewal process framework (*14*), the equation holds the form:

$$j_t = R_t^{(i)} \sum_u i_{t-u}^{nc} \cdot \lambda_u \qquad (13)$$

where λ_u denotes the profile of infectiousness, which is the probability of a secondary transmission from a primary case at time u measured with respect to the symptom onset date.

Notably, u covers both positive and negative integers. Negative integers imply pre-symptomatic transmission, which is characteristic of SARS-CoV-2 infection. The profile of infectiousness λ_u was previously estimated using a gamma distribution shifted 12.3 days to the left that peaked at time zero and indicated 44% of pre-symptomatic infections (*15*).

Because newly infected cases j_t experience symptom onsets that are time-lagged by the incubation period, we can write:

$$E(i_t^{nc}) = \sum_{\tau=1}^{t-1} j_{t-\tau} h_\tau \qquad (14)$$

where h_τ defines the incubation period fitted by a lognormal distribution with a mean of 5.2 days (*16*). Combining equations (13)–(14) results in the renewal equation previously derived by Nakajo and Nishiura (*14*):

$$E(i_t^{nc}) = \sum_{\tau=0}^{t} R_{t-\tau}^{(i)} h_\tau \sum_{u=-x}^{t-\tau} i_{t-\tau-u}^{nc} \lambda_u \qquad (15)$$

where $x = 13$ days is the least integer for the shift of 12.3 days. The observation model was implemented using a negative binomial likelihood function analogous to equation (13), however, $E(i_t^{nc})$ was replaced by a double sum (15).

The effective reproduction number was first modeled by a piecewise constant function of time *t*. The change points were set to every 7 days, which was a rough approximation of both the generation time (*17–19*) and a calendar week. Two alternative approaches were also explored. In the first approach, the change in the effective reproduction number was attributed to PHSMs (Appendix Table 2). In this case, the effective reproduction number remained constant during the intervening time periods but changed abruptly when the PHSMs were implemented. In the second approach, the effective reproduction number was correlated with a change in mobility metrics and involved a sigmoidal, monotonically decreasing change in the baseline (basic) reproduction number, for example, owing to behavioral changes or more efficient case finding and contact tracing toward the end of the epidemic in August–September 2021. The effect of overall mobility reduction was modeled by a combination of mobility metrics in different settings. In this case, the effective reproduction number changed on the log scale according to the following equation:

$$\log R_t^{(i)} = \log R_{0,t} + \sum_{p=1}^{P} \beta_p X_t^{(p)} \qquad (16)$$

where the intercept $\log R_{0,t}$ is the logarithm of the baseline reproduction number given by a sigmoidal function over time (*20*):

$$R_{0,t} = R_0 \left(\eta + \frac{1-\eta}{1+\exp(\xi(t-t_{sw}-v))} \right) \quad (17)$$

where each of five parameters, R_0, η, v, ξ, and t_{sw}, were assigned to a weakly informative prior. The parameter R_0 defined the baseline reproduction number at the beginning of the epidemic. The parameter η measured the reduction in transmission during the later stage of the epidemic, $\eta \sim \text{Beta}(2.5, 4)$, v measured the delay in the reduction of the baseline reproduction number, $v \sim \text{Exponential}(1/5)$, ξ measured the slope of the sigmoidal function, which was restricted to be negative, $\xi \sim \text{Uniform}(0.5, 1.5)$, and t_{sw} defined the change point of the baseline reproduction number during late stage of the epidemic, $t_{sw} \sim \text{Uniform}(\text{July } 1, 2021, \text{August } 25, 2021)$. The variable $X_t^{(p)}$ defined the pth mobility metric on day t across the total $P = 6$ metrics.

The restricted number of mobility metrics $P_{\min} \leq P$ was identified by comparing the "leave-one-out information criterion" LOOIC values for the respective models with the total $P_{\min}$ metrics and $C_P^{P_{\min}}$ combinations among all P metrics. The LOOIC is commonly used in Bayesian frameworks for model selection (*21*). Specifically, the fit of the model with only $P_{\min}$ mobility metrics was compared to the fit of other models by selecting the model with the smallest LOOIC value. The negative binomial likelihood used for the data fit was analogous to (11) and was sequentially replaced by the gamma likelihood (12).

Counterfactual scenarios

The statistical model for the effective reproduction number based on mobility metrics was applied to predict the incidence of COVID-19 under different counterfactual scenarios. Compared with the baseline scenario shown in Appendix Figure 1, panel A, the counterfactual scenarios described situations in which a set of Level 3 measures, initially introduced on May 15, 2021, was shifted either to the right or to the left by y days. This was modeled by accordingly shifting the mobility patterns starting from May 15, 2021. When the mobility patterns were shifted to the left (i.e., earlier implementation of PHSMs), the mobility metrics during the period of (May 15, 2021 – y days) to (May 15, 2021 – 1 day) were replaced with the shifted mobility metrics starting from May 15. Owing to the shift, the final (y – 1) days before the ending time T

remained unfilled. They were replaced with zeros because they had a negligible effect on the results of the counterfactual scenario. When the mobility metrics were shifted to the right (i.e., a later implementation of PHSMs), the unfilled gap in metrics covering the time period from May 15, 2021 through (May 15, 2021 – (y – 1) days) was filled with mobility readings randomly imputed from a 2-week period between May 1 and May 14. For instance, if the gap was 3 days (y = 3), the mobility metrics for those 3 days were imputed by randomly selecting 3 days from May 1–14. The result of each counterfactual scenario was expressed as a cumulative number of infections observed as of August 14, 2021. This cutoff point was chosen because it marked a 3-month period after the initial Level 3 implementation on May 15.

The following protocol was used to perform computer simulations. The seeded number of infections j_0 and the seeding time t_0 were chosen based on a grid search and calculation of either the root-mean-square error (RMSE) or Dawid–Sebastiani score (DSS) (*22*,*23*). Prior to the seeding time, all infections were set to zero. Because the transmission chains observed in April 2021 were thoroughly investigated by Taiwanese authorities, we assumed that the seeding time was between April 5 and April 20, 2021 and the number of seeded infections was unlikely to exceed five. The RMSE value was derived by comparing the simulated incidence $\tilde{\imath}_t$ characterized by the symptom onset date and the nowcasted incidence i_t^{nc} according to the following formula:

$$RMSE(i_t^{nc}, \tilde{\imath}_t) = \left(\sum_{t=t_{min}}^{t_{max}} \frac{(i_t^{nc} - \tilde{\imath}_t)^2}{K}\right)^{1/2} \quad (18)$$

$$DSS(i_t^{nc}, \tilde{\imath}_t) = \left(\frac{i_t^{nc} - \mu_{\mathcal{P}t}}{\sigma_{\mathcal{P}t}}\right)^2 + 2\log\sigma_{\mathcal{P}t} \quad (19)$$

where $K = t_{max} - t_{min} + 1$ is the total number of data points from t_{min} to t_{max} covering the observed time period. t_{min} was set to April 21, 2021 (1 day after the end of the seeding time period), t_{max} was set to August 14, 2021, as discussed earlier. The DSS value representing a proper scoring rule was calculated by comparing the nowcasted counts with the posterior predictive distribution $\mathcal{P}_t$. Denoting the mean of the posterior distribution as $\mu_{\mathcal{P}t}$ and the standard deviation as $\sigma_{\mathcal{P}t}$, we can write:

The model with smaller values of DSS or RMSE was preferred over the others.

To predict the case counts $\tilde{\imath}_t$ and their posterior distribution $\mathcal{P}_t$ for a given counterfactual or baseline scenario, the following two-step procedure was implemented. In the first step, the number of infections $\tilde{\jmath}_t$ was obtained using the renewal process and the Poisson count model:

$$\tilde{\jmath}_t \sim \text{Poisson}\big(E(\tilde{\jmath}_t)\big)$$

$$E(\tilde{\jmath}_t) = R_t^{(i)} \sum_{\tau=1}^{t-1} \tilde{\jmath}_{t-\tau} \cdot \tilde{g}_\tau \qquad (20)$$

for any $t > t_0$. Otherwise, $\tilde{\jmath}_{t=t_0} = j_0$ and $\tilde{\jmath}_{t<t_0} = 0$. The effective reproduction number $R_t^{(i)}$ was defined by equation (16) and included modified mobility metrics according to the chosen counterfactual scenario. The distribution $\tilde{g}_\tau$ defined the generation time distribution that was previously estimated by the gamma distribution with a mean (±SD) of 5.7 ± 1.7 days (*18*). Currently, there is no evidence that the generation time intervals were substantially different between the Alpha variant and the wild-type strain of SARS-CoV-2 (*19*). In the second step, the simulated case counts $\tilde{\imath}_t$ were obtained by combining multinomial samples from the days preceding t, where the size parameter was equal to the number of infections and the probability distribution was given by the incubation period h:

$$\tilde{\imath}_t = \sum_{u>0} z_t^{(t-u)}$$

$$z_{\{s+1,s+2,\dots\}}^{(s)} \sim \text{Multinomial}(\text{size} = \tilde{\jmath}_s, \text{prob.} = \{h_\tau, \tau = 1,2,\dots\}) \qquad (21)$$

Summary

Our framework has several important implications to advance the methods used for the real-time estimation of epidemiological parameters. First, we estimated a time-varied reporting delay distribution that measured the time between symptom onset and case confirmation. Similar to previous works (*3,24,25*), we performed statistical inference within a Bayesian framework and conducted Bayesian nowcasting of cases not yet reported. Our approach was based on using cubic B-splines, which is less computationally demanding than Gaussian processes (*3*). Our method shows a similar performance to approximating the reporting delay by a piecewise constant function, but it forces the coefficient of variation to remain constant (Appendix Figure 3). It appeared that the method was robust to varying the parameters (Appendix Figure 2). Second, we statistically inferred the effective reproduction number according to the date of infection, as proposed by Nakajo and Nishiura (*14*). By using a Bayesian model, we

demonstrated reliance on both pointwise and Bayesian posterior estimates while expanding the original methodology. Our methods improve the real-time estimation techniques and can be used to build accurate risk assessments and to precisely monitor the disease spread at different locations in the future.

Technical details

All model parameters were estimated by using Markov chain Monte Carlo methods within a Bayesian framework. Statistical inference was performed using the *Stan* programming language (*cmdStan* version 2.28.2). Data processing, analysis and presentation of the results were performed using *R* (version 4.1.2) and *Python* (version 3.9) with other base packages involved. The DSS and RMSE values were calculated using the *R* package *scoringutils* (*26*). The code scripts necessary for the replication of our results is available at: https://github.com/aakhmetz/COVID19-Reff-Taiwan-2021.

References

1. Nishiura H, Klinkenberg D, Roberts M, Heesterbeek JA. Early epidemiological assessment of the virulence of emerging infectious diseases: a case study of an influenza pandemic. PLoS One. 2009;4:e6852. PubMed https://doi.org/10.1371/journal.pone.0006852

2. Munayco C, Chowell G, Tariq A, Undurraga EA, Mizumoto K. Risk of death by age and gender from CoVID-19 in Peru, March-May, 2020. Aging (Albany NY). 2020;12:13869–81. PubMed https://doi.org/10.18632/aging.103687

3. Hawryluk I, Hoeltgebaum H, Mishra S, Miscouridou X, Schnekenberg RP, Whittaker C, et al. Gaussian process nowcasting: application to COVID-19 mortality reporting. Preprint at *arXiv*. 2021 https://arxiv.org/abs/2102.11249

4. Kharratzadeh M. Splines in Stan. *Stan Case Studies* 2017, 4. https://mc-stan.org/users/documentation/case-studies/splines_in_stan.html

5. Park SW, Sun K, Champredon D, Li M, Bolker BM, Earn DJD, et al. Forward-looking serial intervals correctly link epidemic growth to reproduction numbers. Proc Natl Acad Sci U S A. 2021;118:e2011548118. PubMed https://doi.org/10.1073/pnas.2011548118

6. Tsuzuki S, Lee H, Miura F, Chan YH, Jung SM, Akhmetzhanov AR, et al. Dynamics of the pneumonic plague epidemic in Madagascar, August to October 2017. Euro Surveill. 2017;22:2–7. PubMed https://doi.org/10.2807/1560-7917.ES.2017.22.46.17-00710

7. Nishiura H, Linton NM, Akhmetzhanov AR. Serial interval of novel coronavirus (COVID-19) infections. Int J Infect Dis. 2020;93:284–6. PubMed https://doi.org/10.1016/j.ijid.2020.02.060

8. Ryu S, Kim D, Lim JS, Ali ST, Cowling BJ. Serial interval and transmission dynamics during SARS-CoV-2 Delta variant predominance, South Korea. Emerg Infect Dis. 2022;28:407–10. PubMed https://doi.org/10.3201/eid2802.211774

9. Greene SK, McGough SF, Culp GM, Graf LE, Lipsitch M, Menzies NA, et al. Nowcasting for real-time COVID-19 tracking in New York City: an evaluation using reportable disease data from early in the Pandemic. JMIR Public Health Surveill. 2021;7:e25538. PubMed https://doi.org/10.2196/25538

10. Counotte MJ, Althaus CL, Low N, Riou J. Impact of age-specific immunity on the timing and burden of the next Zika virus outbreak. PLoS Negl Trop Dis. 2019;13:e0007978. PubMed https://doi.org/10.1371/journal.pntd.0007978

11. Nouvellet P, Bhatia S, Cori A, Ainslie KEC, Baguelin M, Bhatt S, et al. Reduction in mobility and COVID-19 transmission. Nat Commun. 2021;12:1090. PubMed https://doi.org/10.1038/s41467-021-21358-2

12. Li M, Dushoff J, Bolker BM. Fitting mechanistic epidemic models to data: A comparison of simple Markov chain Monte Carlo approaches. Stat Methods Med Res. 2018;27:1956–67. PubMed https://doi.org/10.1177/0962280217747054

13. Gostic KM, McGough L, Baskerville EB, Abbott S, Joshi K, Tedijanto C, et al. Practical considerations for measuring the effective reproductive number, Rt. PLOS Comput Biol. 2020;16:e1008409. PubMed https://doi.org/10.1371/journal.pcbi.1008409

14. Nakajo K, Nishiura H. Estimation of R(t) based on illness onset data: An analysis of 1907-1908 smallpox epidemic in Tokyo. Epidemics. 2022;38:100545. PubMed https://doi.org/10.1016/j.epidem.2022.100545

15. He X, Lau EHY, Wu P, Deng X, Wang J, Hao X, et al. Temporal dynamics in viral shedding and transmissibility of COVID-19. Nat Med. 2020;26:672–5. PubMed https://doi.org/10.1038/s41591-020-0869-5

16. Li Q, Guan X, Wu P, Wang X, Zhou L, Tong Y, et al. Early transmission dynamics in Wuhan, China, of novel coronavirus–infected pneumonia. N Engl J Med. 2020;382:1199–207. PubMed https://doi.org/10.1056/NEJMoa2001316

17. Ganyani T, Kremer C, Chen D, Torneri A, Faes C, Wallinga J, et al. Estimating the generation interval for coronavirus disease (COVID-19) based on symptom onset data, March 2020. Euro Surveill. 2020;25:2000257. PubMed https://doi.org/10.2807/1560-7917.ES.2020.25.17.2000257

18. Lau, Y.C., Tsang, T.K., Kennedy-Shaffer, L., Kahn, R., Lau, E., et al. Joint estimation of generation time and incubation period for coronavirus disease (Covid-19). J. Infect. Dis. 2021;jiab424.

19. Linton NM, Akhmetzhanov AR, Nishiura H. Correlation between times to SARS-CoV-2 symptom onset and secondary transmission undermines epidemic control efforts. medRxiv. 2021 Aug 31. https://doi.org/10.1101/2021.08.29.21262512

20. Grinsztajn L, Semenova E, Margossian CC, Riou J. Bayesian workflow for disease transmission modeling in Stan. Stat Med. 2021;40:6209–34. PubMed https://doi.org/10.1002/sim.9164

21. Vehtari A, Gelman A, Gabry J. Practical Bayesian model evaluation using leave-one-out cross-validation and WAIC. Stat Comput. 2017;27:1413–32. https://doi.org/10.1007/s11222-016-9696-4

22. Dawid AP, Sebastiani P. Coherent dispersion criteria for optimal experimental design. Ann Stat. 1999;27:65–81. https://doi.org/10.1214/aos/1018031101

23. Funk S, Camacho A, Kucharski AJ, Lowe R, Eggo RM, Edmunds WJ. Assessing the performance of real-time epidemic forecasts: A case study of Ebola in the Western Area region of Sierra Leone, 2014-15. PLOS Comput Biol. 2019;15:e1006785. PubMed https://doi.org/10.1371/journal.pcbi.1006785

24. Mallela A, Neumann J, Miller EF, Chen Y, Posner RG, Lin YT, et al. Bayesian Inference of state-level COVID-19 basic reproduction numbers across the United States. Viruses. 2022;14:157. PubMed https://doi.org/10.3390/v14010157

25. McGough SF, Johansson MA, Lipsitch M, Menzies NA. Nowcasting by Bayesian Smoothing: A flexible, generalizable model for real-time epidemic tracking. PLOS Comput Biol. 2020;16:e1007735. PubMed https://doi.org/10.1371/journal.pcbi.1007735

26. Bosse NI, Abbott S. EpiForecasts, Funk, S. scoringutils: utilities for scoring and assessing predictions. *Zenodo* 2020. https://doi.org/10.5281/zenodo.4618017.

Appendix Table 1. List of infector-infectee pairs from a series of epidemiological investigations conducted by Taiwan Centers for Disease Control from the beginning of 2020 through March 2021 used for estimation of the serial interval

#	Infector National ID Case #	Infector Onset Date	Infectee National ID Case #	Infectee Onset Date	Classification*	Note
1	5	2020-01-25	8	2020-01-26	certain	
2	10	2020-01-21	9	2020-01-27	certain	
3	19	2020-01-27	21	2020-02-06	probable	family cluster
4	19	2020-01-27	22	2020-01-28	probable	family cluster
5	19	2020-01-27	23	2020-02-03	probable	family cluster
6	24	2020-01-22	25	2020-01-24	probable	family cluster
7	27	2020-02-05	28	2020-01-31	probable	family cluster, transmission chain is not clear
8	27	2020-02-05	29	2020-01-29	probable	family cluster, transmission chain is not clear
9	27	2020-02-05	30	2020-02-04	probable	family cluster, transmission chain is not clear
10	27	2020-02-05	32	2020-02-24	probable	a caretaker, transmission chain is not clear
11	34	2020-02-18	36	2020-02-18	certain	a nurse
12	34	2020-02-18	37	2020-02-25	certain	a nurse
13	34	2020-02-18	38	2020-02-25	certain	a nurse
14	34	2020-02-18	46	2020-03-03	probable	son, family cluster
15	39	2020-02-20	43	2020-03-03	certain	
16	122	2020-03-18	307	2020-03-25	certain	
17	71	2020-03-13	92	2020-03-17	certain	
18	59	2020-03-12	103	2020-03-15	certain	
19	59	2020-03-12	130	2020-03-17	certain	
20	160	2020-03-14	124	2020-03-17	probable	
21	160	2020-03-14	168	2020-03-16	probable	
22	160	2020-03-14	169	2020-03-18	probable	
23	84	2020-03-16	216	2020-03-20	certain	
24	228	2020-03-21	247	2020-03-23	certain	
25	209	2020-03-21	246	2020-03-23	certain	
26	289	2020-03-22	293	2020-03-23	certain	
27	290	2020-03-22	335	2020-03-23	certain	
28	277	2020-03-22	269	2020-03-23	certain	
29	269	2020-03-23	299	2020-03-26	certain	
30	336	2020-03-17	347	2020-03-24	certain	
31	301	2020-03-06	352	2020-03-30	certain	
32	356	2020-03-17	343	2020-03-20	certain	
33	356	2020-03-17	365	2020-03-25	certain	
34	812	2020-12-29	838	2021-01-08	certain	
35	838	2021-01-08	839	2021-01-09	certain	
36	839	2021-01-09	870	2021-01-18	certain	
37	838	2021-01-08	869	2021-01-16	certain	
38	838	2021-01-08	852	2021-01-14	certain	
39	838	2021-01-08	856	2021-01-16	certain	
40	856	2021-01-16	863	2021-01-14	probable	
41	856	2021-01-16	868	2021-01-17	probable	
42	863	2021-01-14	864	2021-01-14	probable	family cluster
43	863	2021-01-14	865	2021-01-18	probable	family cluster
44	863	2021-01-14	907	2021-01-28	probable	family cluster
45	863	2021-01-14	910	2021-01-29	probable	family cluster
46	765	2020-12-12	771	2020-12-14	certain	
47	765	2020-12-12	760	2020-12-16	certain	
48	765	2020-12-12	766	2020-12-17	certain	
49	852	2021-01-14	881	2021-01-21	certain	
50	870	2021-01-18	924	2021-02-01	probable	
51	889	2021-01-19	890	2021-01-20	probable	
52	889	2021-01-19	908	2021-01-29	probable	

*The pair was classified as certain if the epidemiological link between an infector and an infectee could be established on a one-to-one relationship based on cumulative evidence. The pair was classified as probable if more than one possible infector could be assigned to a given infectee.

Appendix Table 2. Criteria and prevention measures of 4-level COVID-19 alert system in Taiwan

Level	Epidemic level as criteria	Prevention measures
Level 1	Sporadic community infections caused by imported cases	• Masks must be worn all the time while using public transportation and being at crowded public venues.<br>• Recommendation to cancel or postpone non-essential gatherings that will bring people into close contact with each other.<br>• Business and public venues must implement prevention measures which include an identification-based registration system, social distancing, temperature checks, and routine disinfection.
Level 2	New local cases with unknown source of infection	• Impose fines on those who do not obey the regulation of mask wearing.<br>• Suspend all outdoor gatherings with 500+ participants and indoor gatherings with 100+ participants.<br>• Any other gatherings must implement prevention measures including social distancing, mask wearing/partitions, an identification-based registration system, temperature checks, routine disinfection, crowd control, and crowd mobility management.<br>• Business venues must impose crowd control; those that are unable to implement necessary epidemic prevention measures must temporarily suspend operations.<br>• If necessary, entertainment related business and public venues are asked to be closed.
Level 3	3+ community clusters in one week or 10+ local cases with unknown source of infection	• Masks must be worn at all times outdoors.<br>• Suspend all outdoor gatherings with 10+ participants and indoor gatherings with 5+ participants.<br>• Apart from essential services, which include maintenance venues, medical and public services, all other business and public venues must be closed.<br>• In the community with an ongoing active transmission where the rapid containment is required, residents must comply with COVID-19 testing protocols, do not leave the pre-defined control places and also suspend all gatherings and close schools.
Strengthened level 3		Five additional measures on top of that in Level 3:<br>• Violation of mask wearing regulation at all times will be fined.<br>• Strict inspection of entertainment related venues which have already been announced to be closed. Illegal operation, which involves operators, on-site practitioners, consumers and participants will be penalized accordingly with the law.<br>• Only take-out for all food service is possible. The enhanced crowd control is required for supermarkets and all other markets.<br>• Cancellation of all banquets for wedding ceremonies and public memorial ceremonies for funerals.<br>• Suspension of all religious gatherings. Religious venues must be closed for the public.
Level 4	Rapid increase of local cases (average number of cases more than 100 per day within 14 days) and half of them with unknown source of infection	• Residents can leave their home only for essential activities, for example, to buy food, receive medical treatment, or to do essential work. The social distance must be maintained and wearing a mask must be done at all times outdoors.<br>• Residents must wear a mask and maintain social distance when at home.<br>• Suspension of all gatherings.<br>• Apart from essential services, which include maintenance venues, necessary medical and public services, all other venues and schools must be closed.<br>• Implementation of a lockdown policy in cities/counties or districts that reported severe outbreaks. The lockdown areas must be defined precisely and clearly, which will include the definition of restriction zones for people's entrance and exit. Residents must stay home.

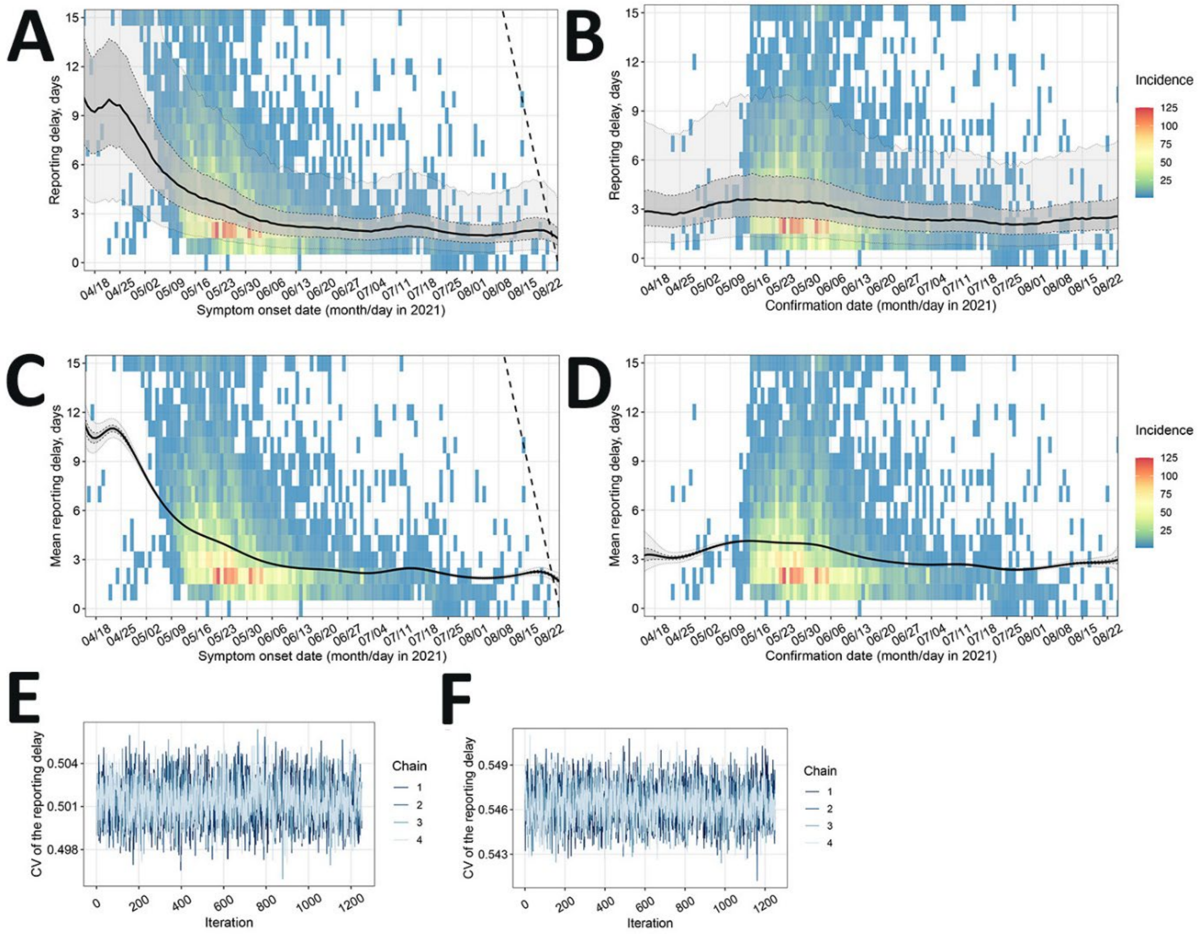

Appendix Figure 1. Data from a study of transmission dynamics and effectiveness of control measures during a COVID-19 surge, Taiwan, April-August 2021. Posterior distribution of the forward (by symptom onset date [A]) and backward (by confirmation date [B]) reporting delay, which also involved estimation of the mean (C,D) and coefficient of variation (CV) (E,F) of the reporting delay. The heatmap in the background shows the incidence of COVID-19 cases by a 2-dimensional contingency table, with horizontal axis being a date of symptom onset (A,C,E) or case confirmation (B,D,F) and with vertical axis being the observed reporting delay. The color code is indicated in the legend. CV remained constant during the epidemic, and it is shown by a trace plot of Markov chain Monte Carlo (MCMC) simulations. The dashed black line indicates the right truncation corresponding to the latest update of August 25, 2021.

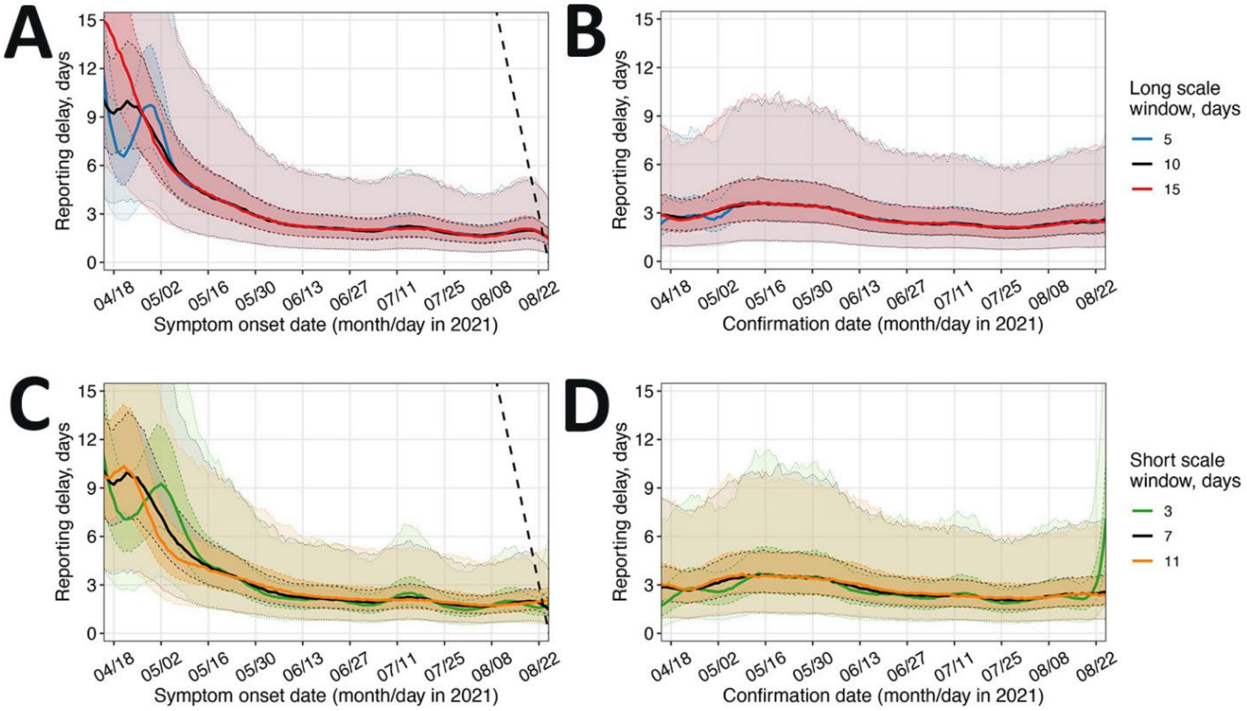

Appendix Figure 2. Data from a study of transmission dynamics and effectiveness of control measures during a COVID-19 surge, Taiwan, April-August 2021. Effect of varying the long-scale time window W (A,B) and short-scale time window ω (C,D) on the fit of the forward (A,C) and backward (B,D) reporting delay distributions. Only 1 of 2 parameters was varied, while the other was fixed at its baseline value—10 days for the long scale window and 7 days for the short-scale window—and shown in black (legend). The dashed black line in AC indicates the right truncation line respectively to the latest update date of August 25, 2021.

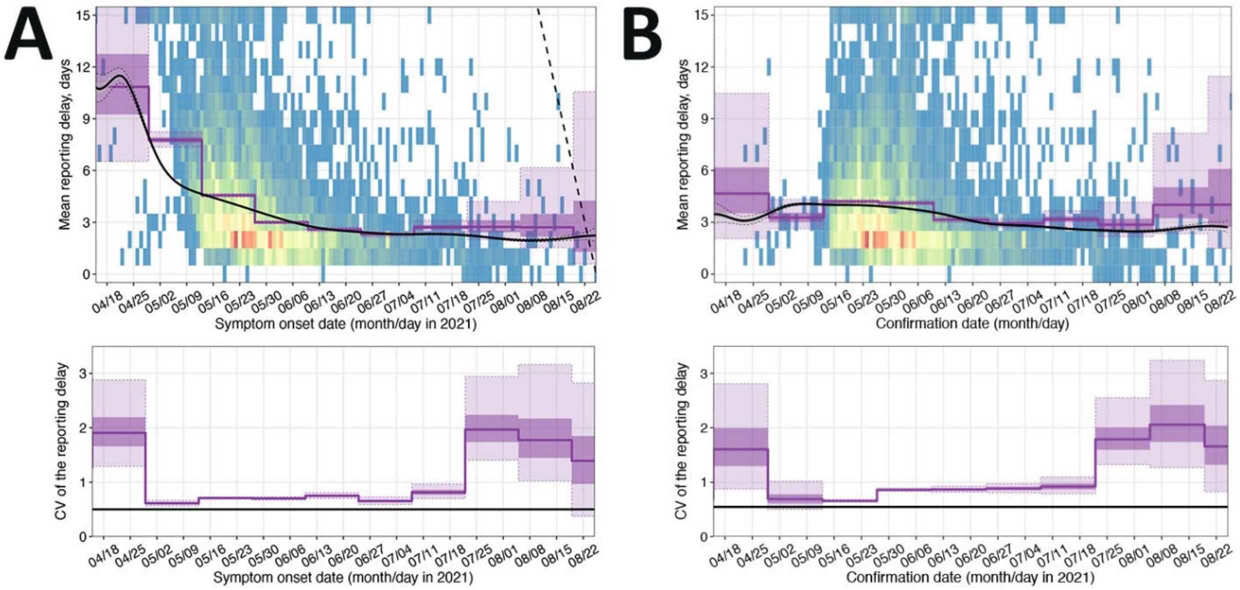

Appendix Figure 3. Data from a study of transmission dynamics and effectiveness of control measures during a COVID-19 surge, Taiwan, April-August 2021. Comparing the mean and coefficient of variation (CV) for the forward (A) and backward (B) reporting delay distributions when the mean is given by a cubic B-spline and CV is constant (black), or both the mean and CV are given by a piecewise-constant function over time with time-window of 14 days (purple). The heatmap shows the incidence of COVID-19 cases represented as a 2-dimensional contingency table, where the horizontal axis is the date of symptom onset or case confirmation and the vertical axis is the reporting delay. The color code is shown in legend. CV was constant during the epidemic and shown as a trace plot of MCMC simulations. The dashed black line in A (top panel) indicates the right truncation line respectively to the latest update date of August 25, 2021.

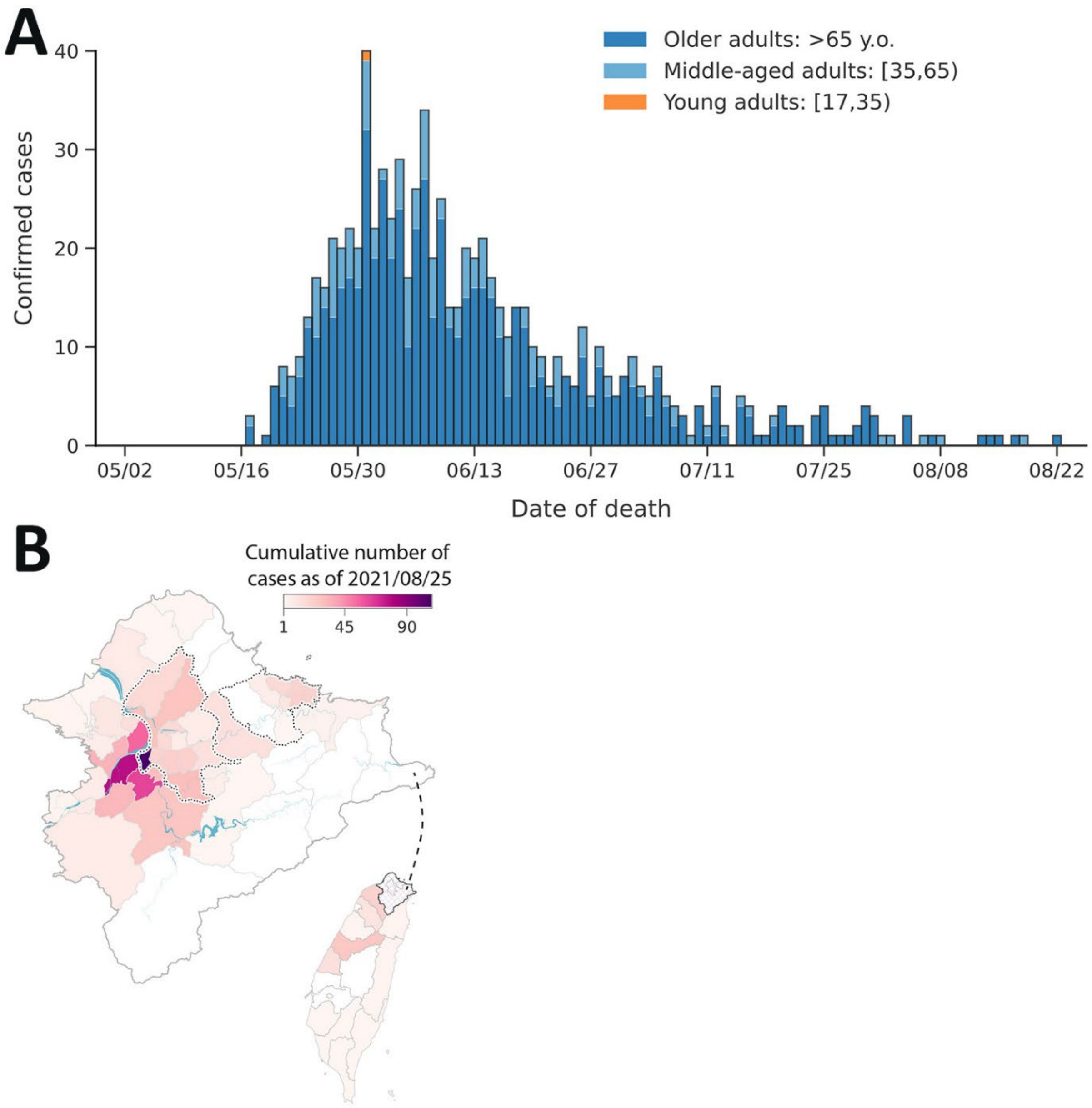

Appendix Figure 4. Data from a study of transmission dynamics and effectiveness of control measures during a COVID-19 surge, Taiwan, April-August 2021. Age and spatial distribution of deaths confirmed in Taiwan from May through August 25, 2021. A) Epidemiologic curve of confirmed COVID-19 death cases stratified by age group and shown by date of death. B) Geographic distribution of deaths. The colormap indicates the cumulative number of deaths reported as of August 25, 2021, at the district level for Taipei City, New Taipei City, and Keeling City, and at county level for the rest of Taiwan.

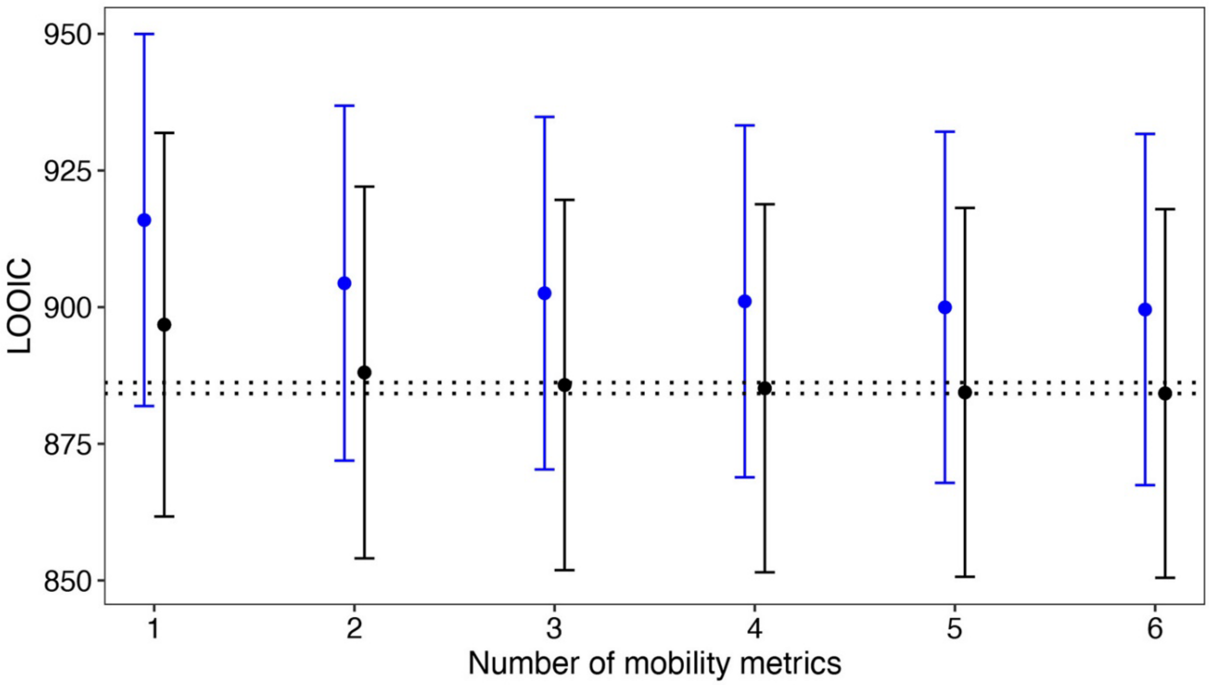

Appendix Figure 5. Data from a study of transmission dynamics and effectiveness of control measures during a COVID-19 surge, Taiwan, April-August 2021. Comparing the "leave-one-out" information criterion (LOOIC) values for 2 models when the baseline reproduction number is constant during the epidemic (blue) or modeled by a sigmoidal monotonically decreasing function of time implying one changepoint (black). The double-dashed line indicates LOOIC values exceeding the minimal LOOIC value by no more than 2 units.

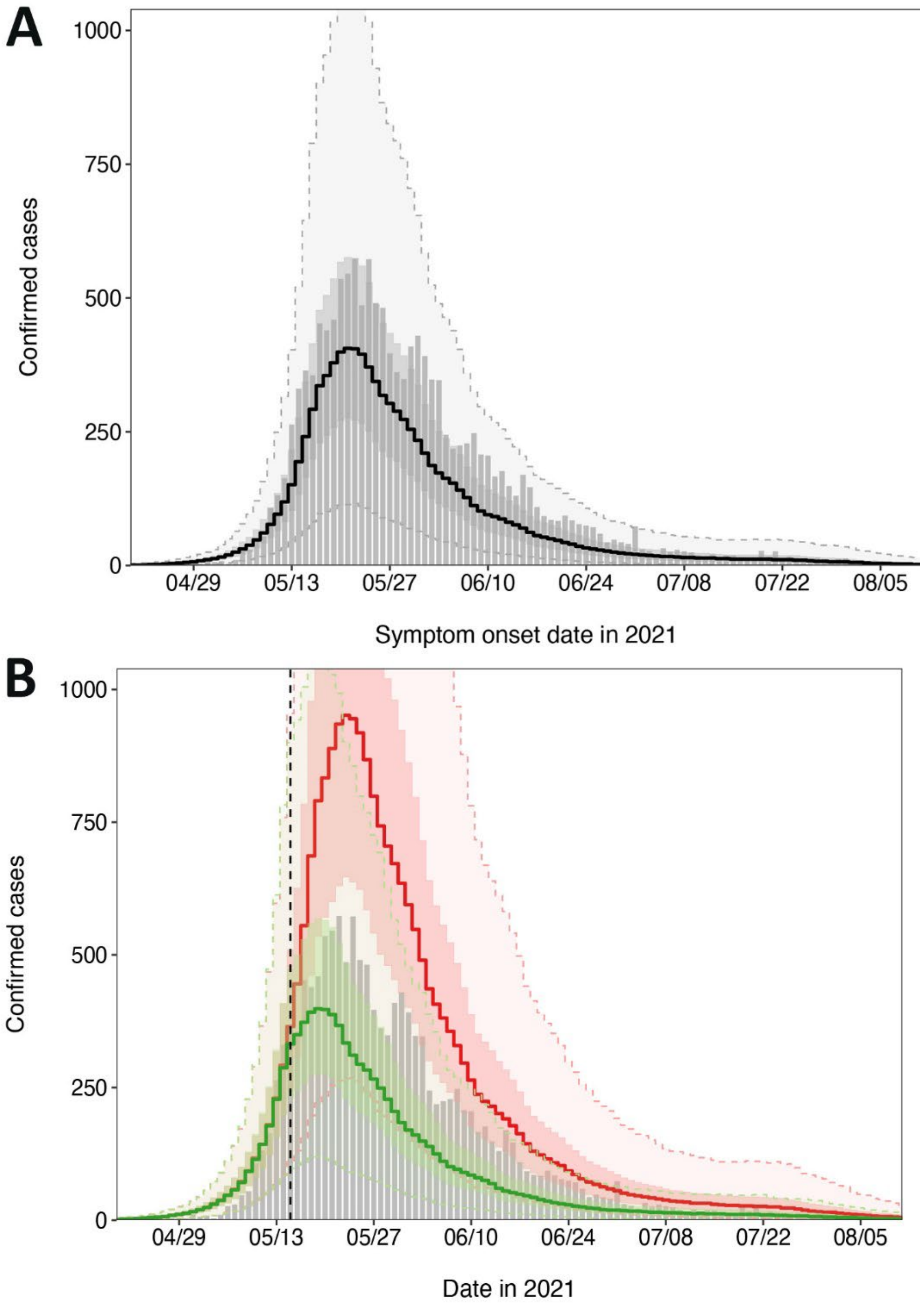

Appendix Figure 6. Data from a study of transmission dynamics and effectiveness of control measures during a COVID-19 surge, Taiwan, April-August 2021. Reconstructed epidemiologic curve for baseline scenario (A) and 2 counterfactual scenarios (B) when the Level 3 measures were implemented either 3 days earlier (green) or 3 days later (red). The thick line indicated the median of the posterior distribution. The light shaded area indicates the 95% credible interval, whereas the dark shaded area shows the interquartile range of the posterior distribution. Gray bars show the observed and backprojected counts of confirmed cases by date of symptom onset. The vertical dashed line in B indicates the date of May 15, 2021, when the baseline Level 3 measures were implemented at national level.
